# A new species of *Leptopulvinaria* Kanda from China, with a key to species (Hemiptera, Coccomorpha, Coccidae)

**DOI:** 10.3897/zookeys.781.25713

**Published:** 2018-08-13

**Authors:** Xiaoying He, Yangyang Han, Sanan Wu

**Affiliations:** 1 Key Laboratory for Silviculture and Conservation of Ministry of Education, Beijing Forestry University, Beijing 100083, China Beijing Forestry University Beijing China; 2 Shanghai Forestry Station, Shanghai 200072, China Shanghai Forestry Station Shanghai China

**Keywords:** China, Pulvinariini, soft scale insect, taxonomy

## Abstract

A new species *Leptopulvinariasapinda***sp. n.** is described and illustrated based on adult females collected on *Sapindussaponaria* (Sapindaceae) from Shanghai and Jiangsu. This is the first report of *Leptopulvinaria* species in China. A key to the species of *Leptopulvinaria* Kanda is provided.

## Introduction

The family Coccidae (Hemiptera: Sternorrhyncha: Coccomorpha) is the third largest family of the Coccomorpha after the Diaspididae (armored scales) and Pseudococcidae (mealybugs), consisting of 1185 described species, distributed in 169 genera all over the world (García Morales et al. 2018, Williams and Hodgson 2014). Species belonging to this family are widespread throughout the world and many of them are important pests on agricultural, horticultural, and ornamental plants (Henderson and Hodgson 2005). These include *Pulvinariasalicicola* (Borchsenius), which caused much damage to growth of roadside afforested willows in Lindian County, Heilongjiang Province of China with an injury rate as high as 90% (Zhang et al. 1993), and *Ceroplastesjaponicus* (Green), which can cause deformation or death of poplars and the percentage of damaged trees is more than 65% in some areas of Lianyungang city, Jiangsu Province (Wang et al. 2008).

The genus *Leptopulvinaria* was established by Kanda (1960), based on its type species, *Leptopulvinariaelaeocarpi* Kanda, 1960, and it was diagnosed by absence of tarsal and claw digitules. Tang (1991) placed this genus in the tribe Pulvinariini. Hodgson (1994) redescribed the type species based on the type specimen and found that it did in fact have tarsal digitules and claw digitules. He did not affirm the exact taxonomic positon of the species, since it is difficult to ascertain the exact distribution of the dorsal and ventral tubular ducts owing to poor condition of type specimens. Recently, Tanaka and Amano (2008) redescribed *L.elaeocarpi* based on newly collected material and described the second species *L.kawaii* Tanaka & Amano from Japan. They also observed the production of ovisacs of both these species at their oviposition periods and clearly and definitely placed this genus in the tribe Pulvinariini. Choi and Lee (2017) reported *L.kawaii* from South Korea. Currently, species of this genus are therefore known only from Japan and South Korea and the genus includes only two species, namely *Leptopulvinariaelaeocarpi* Kanda and *Leptopulvinariakawaii* Tanaka & Amano.

In the course of our taxonomic study of soft scales (Coccidae) in China, we found an undescribed species which clearly belongs to this genus. Here, we describe and illustrate adult female specimens of this new species. This report is the first formal record of the occurrence of *Leptopulvinaria* species in China, and it may be useful for further taxonomic and biogeographic study of the genus and its species. A key to all three species of *Leptopulvinaria* is provided.

## Materials and methods

Slide mounting methods for the specimens in this study followed Hodgson and Henderson (2000). Terminology of the morphological features used in the description mainly followed Kondo and Hodgson (2013) and Tanaka and Kondo (2015), who avoided using the term “pregenital disc-pores” or “perivulvar pores”, because in several species of Coccidae, these pores are not restricted to the pregenital or perivulvar area, and can be present throughout the medial area of the venter; so that using the term “pregenital” or “perivulvar” may be misleading. The species described in this study also have the pores not only in the pregenital area but also on the medial area of the venter. The term “multilocular pore” is therefore used herein for the pores with multiple loculi, with the exception of spiracular pores. The mounted specimens were examined under a compound light microscope (Leica DME) fitted with an ocular micrometer. All measurements were given (minimum-maximum range) in micrometers (µm), except for body length and width which are given in millimeters (mm).

All specimens are deposited in the Insect Collection, the Department of Forestry Protection, Beijing Forestry University, Beijing, China (**BFUC**).

## Taxonomy

### Genus Leptopulvinaria

Taxon classificationAnimaliaHemipteraCoccidae

Kanda, 1960

#### Type species.

*Leptopulvinariaelaeocarpi* Kanda, 1960, by monotypy and original designation.

#### Generic diagnosis.

**Adult female.** Body elongate oval, broadest at thorax or anterior abdomen. *Dorsum*. Derm membranous. Tubular ducts and microducts frequent. Tubercles convex, occasionally absent. *Margin*. Setae spinose, each with a simple pointed apex. Stigmatic clefts not deep but distinct; each with one to four (usually three) stigmatic spines. *Venter*. Antennae with eight or nine (usually eight) segments. Legs each with a well-developed tibio-tarsal articulation and an articulatory sclerosis. Multilocular pores each with nine to eleven loculi, present mainly across most abdominal segments. Spiracular pores each with four to six (usually five) loculi. Two types of tubular ducts present. With one or two pairs of long setae medially on all abdominal and thoracic segments (occasionally lacking on thoracic segments). For further diagnostic characteristics, see Tanaka and Amano (2008).

##### Key to *Leptopulvinaria* species based on slide-mounted adult females’ morphology

**Table:** 

1	Ventral tubular ducts absent medially on head and thoracic segments, though rarely a few may be present	**2**
–	Ventral tubular ducts present medially on head and thoracic segments	***L.kawaii* Tanaka & Amano, 2008**
2	Dorsal tubular ducts, microducts and setae arranged in a reticulate pattern, multilocular pores absent on head and thorax, though occasionally a few present on metathorax, preopercular pores restricted to anterior anal plates	***L.elaeocarpi* Kanda, 1960**
–	Dorsal tubular ducts, microducts and setae not arranged in a reticulate pattern, multilocular pores numerous on head and thorax, preopercular pores extend from anterior anal plates to prothorax	***L.sapinda* sp. n.**

### Leptopulvinaria sapinda sp. n.

Taxon classificationAnimaliaHemipteraCoccidae

http://zoobank.org/46EDC4B0-9BFD-45DC-82FF-6E3134DC0274

#### Material examined.

**Holotype**: Adult female. CHINA, Shanghai City, Qingpu District, 7.vi.2017, on *Sapindussaponaria* L. (Sapindaceae), coll. Yangyang Han, 1♀(BFUC). **Paratypes**: Same data as holotype, 18♀(BFUC); CHINA, Jiangsu Province, Kunshan City, 11.X.2016, on same host as holotype, coll. Lei Gao, 11♀♀(BFUC).

#### Description.

**Figure 1.**
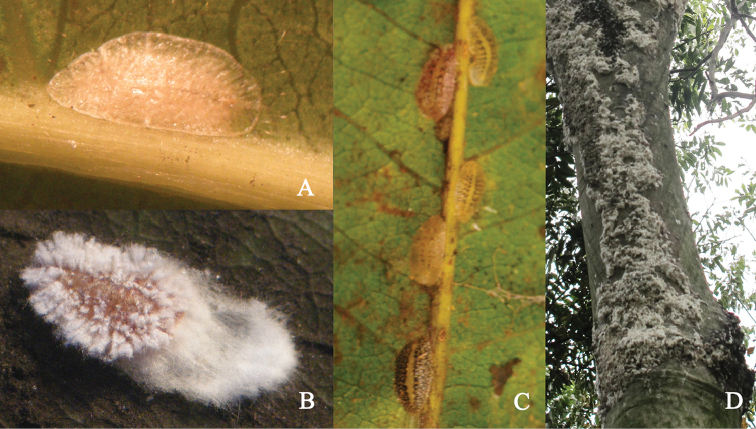
*Leptopulvinariasapinda* sp. n., **A** young adult female **B** adult female after oviposition **C** adult females from young to mature stages **D** ovisacs on trunk of host tree.

**Adult female. Unmounted material**: (Figure 1A–C). Adult female more or less pointed anteriorly, usually somewhat asymmetrical, the young one whitish or light yellowish (Figure 1A), changing to with dark brown reticulations on dorsum except midline, the mature one black, with a longitudinal yellowish stripe along middle line of dorsum (Figure 1C). After oviposition (Figure 1B), the dorsum with wax filaments mainly on the marginal and submarginal area; wax secreted forming a short white ovisac.

#### Mounted material

**Figure 2.**
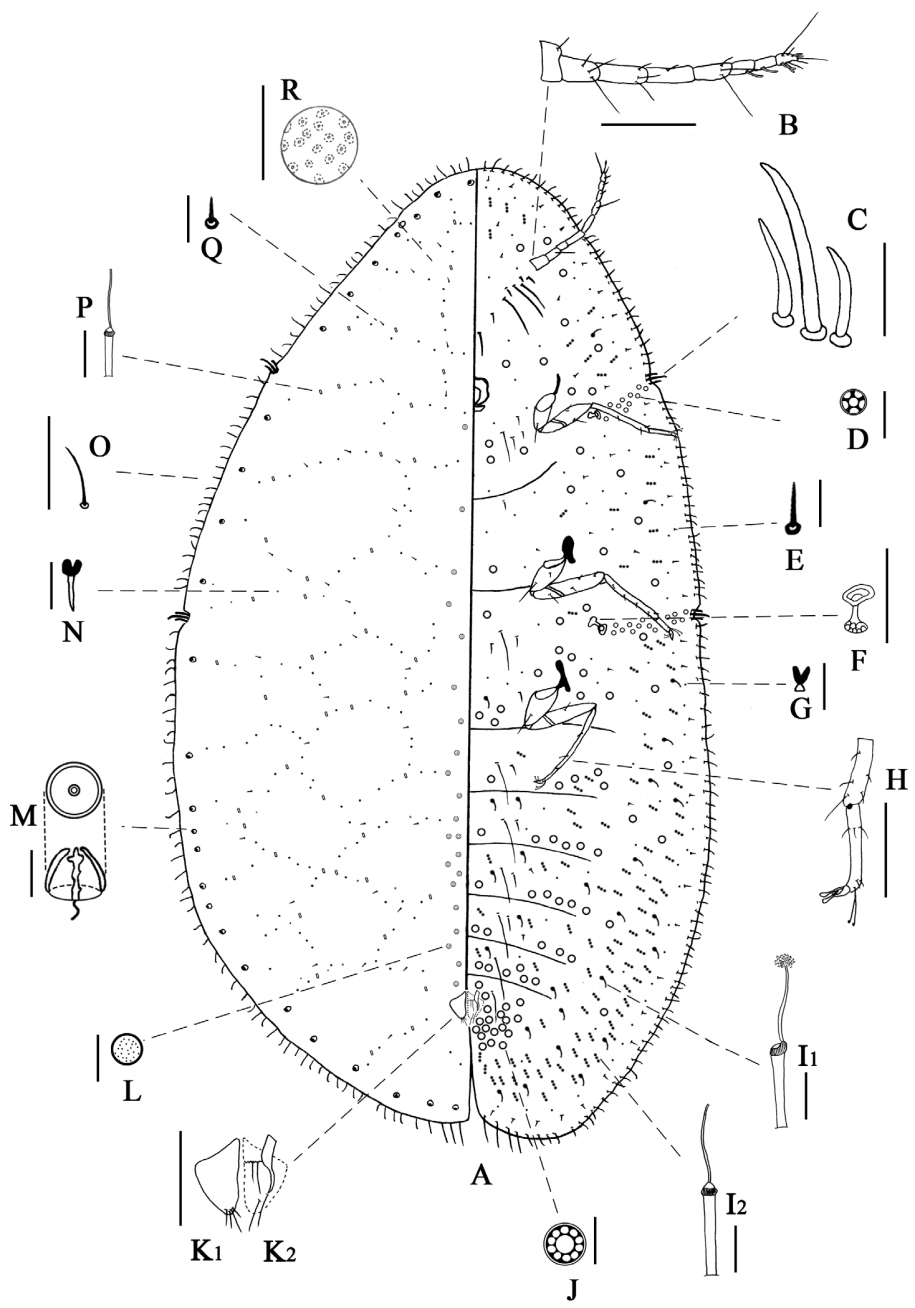
Adult female of *Leptopulvinariasapinda* sp. n., **A** body derm **B** antennae **C** stigmatic spine **D** spiracular pore **E** submarginal seta **F** spiracle **G** ventral microduct **H** leg **I_1_**, **I_2_** ventral tubular duct **J** multilocular pore **K_1_** anal plate **K_2_** ano-genital fold **L** preopercular pore **M** dorsal tubercle **N** dorsal microduct **O** marginal setae **P** dorsal tubular duct **Q** dorsal seta **R** dermal areolation. Scale bars: 200 μm (**B, F, H, K, R**); 20 μm (**C, O**); 10 μm (**D, E, G, I, J, L, N, P, Q** except **A**).

(Figure 2A–R). Body (Figure 2A) elongate oval, 2.2–5.3 mm long, 1.2–3.0 mm wide. Margin with a slight indentation at each stigmatic cleft and sometimes also near each eyespot. Anal cleft 400–770 μm, approximately 1/6–1/7 body length.

#### Dorsum.

Derm membranous. Dermal areolations (Figure 2R) well developed. Dorsal tubercles (Figure 2M) convex, each 11–13 μm in diameter, present on submarginal area, 12–14 between anterior spiracular clefts, 3–5 between each anterior and posterior spiracular clefts, and 10–15 between each posterior spiracular clefts and anal cleft. Preopercular pores (Figure 2L) circular, obvious, each with a diameter of 6–8 µm, present in a small group of 36 to 56 in front of anal plates, extending to prothorax. Tubular ducts (Figure 2P) of one type, outer ductule 8–10 μm long, 2–3 μm wide, inner ductule 12–17 μm long, 1 μm wide, arranged in a reticulate pattern with microducts (Figure 2N). Dorsal setae (Figure 2Q) 6–7 μm long, spiniform, arranged like tubular ducts and microducts. Anal plates (Figure 2K_1_) each triangular, 150–158 μm long, 63–75 μm wide, anterolateral margin slightly concave, 78–100 μm long; posterolateral margin slightly convex, 115–138 μm long. Each plate with four apical setae. Ano-genital fold (Figure 2K_2_) with two pair of setae along anterior margin and two pairs laterally. Anal ring subcircular, with one or two rows of translucent pores and six or eight anal ring setae. Eyespots present near margin.

#### Margin.

Marginal setae (Figure 2O) 14–29 μm long, straight, or slightly curved, rather bluntly pointed; distributed as follow: 60–69 between anterior stigmatic cleft, 21–27 between each anterior and posterior stigmatic cleft, and 52–64 between each posterior stigmatic cleft and anal cleft. Stigmatic clefts (Figure 2C) not deep but distinct; with three (except one with four) stigmatic spines in each cleft; median spine 32–56 μm long, 1.5–2.3 times as long as lateral spines, slightly curved, bluntly pointed; lateral spines slightly curved, bluntly pointed, 14–34 μm long. Eyespots present on margin.

#### Venter.

Derm membranous. Ventral setae: one or two pairs of long setae, 125–238 μm long, present medially on all abdominal and thoracic segments, and also near each coxa (a few pairs of setae occasionally absent on thoracic segments); three pairs of long setae present between antennae, 225–258 μm long; short setae (Figure 2E) 11–14 μm long, slender, acute, mostly straight, and distributed evenly. Antennae (Figure 2B) well developed, 8–segmented, 493–678 μm long, third segment longest; length of segments I to VIII (μm): 60–75, 75–113, 90–150, 78–125, 65–93, 48–83, 38–68, 43–53, respectively; segment VI; VII; VIII each with 1, 1, 4 fleshy setae. Clypeolabral shield 128–168 μm long, 125–168 μm wide. Labium 68–80 μm long, 80–113 μm wide. Legs (Figure 2H) well developed, with a tibio-tarsal articulation and an articulatory sclerosis; claw without denticle; tarsal digitules slender, knobbed; claw digitules broad, and expanded at apex; hind trochanter + femur 275–370 μm long, hind tibia + tarsus 385–558 μm long; claw 35–45 μm long. Ratio of lengths of hind tibia + tarsus to hind trochanter + femur 1.4–1.8. Ratio of lengths of hind tarsus to tibia 1.9–2.3. Anterior spiracles each 68–88 μm long, 45–60 μm wide; posterior spiracles (Figure 2F) each 75–100 μm long, 55–75 μm wide. Spiracular pores (Figure 2D) present in narrow bands one to three pores wide between margin and each spiracle; each mainly with five loculi, 5 μm in diameter, 22–43 in anterior spiracular pore band, 33–56 in posterior spiracular pore band. Multilocular pores (Figure 2J) 7–8 μm in diameter, mainly ten loculi, frequent around anal area, in transverse bands on abdomen, and also scattered in head and thorax. Ventral microducts (Figure 2G) scattered. Ventral tubular ducts (Figure 2I) of two types present: I) a duct (Figure 2I_1_) with a narrow inner ductule and a well-developed terminal gland; outer ductule 10–15 μm long, 3–4 μm wide, inner ductule 16–20 μm long, 1–2 μm wide, 5 μm wide for terminal gland; present submarginally on posterior segments, where they are mixed with type II ducts, a few ducts also present medially on abdominal segments (occasionally present on submarginal head and thorax). II) a duct (Figure 2I_2_) with a slender inner filament; outer ductule 14–16 μm long, 2–3 μm wide, inner ductule 13–18 μm long, 1 μm wide; numerous in submarginal area and mixed with ducts of type I, but becoming sparse on thorax and head, a few present medially on abdominal segments.

#### Distribution.

China (Jiangsu and Shanghai)

#### Host plant.

*Sapindussaponaria* L. (Sapindaceae)

#### Etymology.

The specific epithet is taken from the genus name of host plant.

#### Remarks.

The new species is easily distinguished from the two other *Leptopulvinaria* species by having dorsal tubular ducts, microducts, and setae arranged in a reticulate pattern, and numerous multilocular pores on head and thorax. Moreover, *L.sapinda* sp. n. has a group of preopercular pores extending from anterior anal plates to prothorax, and has 10–15 dorsal tubercles between each posterior stigmatic cleft and the anal cleft, whereas *L.elaeocarpi* has a small group of preopercular pores restricted to anterior anal plates, and 1–4 dorsal tubercles between each posterior stigmatic cleft and the anal cleft. In *L.kawaii*, preopercular pores are absent (or if there are any, then they are difficult to see) and there are only 0–7 dorsal tubercles between each posterior stigmatic cleft and the anal cleft.

During the pre-oviposition period, the adult females of this new species suck plant juices mainly along the main and lateral veins of leaves (Figure 1C). When ovipositing, they usually climb to the trunk and branches (although occasionally they stay on the leaves) to lay eggs (Figure 1B, 1D). Similar behavior, namely changing infesting place on the host trees before ovipositon is also reported in *L.kawaii* (Kawai 1980). This fact may indicate that *L.sapinda* sp. n. is probably close to *L.kawaii* and supports the placement of the new species in this genus.

## Supplementary Material

XML Treatment for Leptopulvinaria

XML Treatment for Leptopulvinaria sapinda
